# Is the relationship between increased knee muscle strength and improved physical function following exercise dependent on baseline physical function status?

**DOI:** 10.1186/s13075-017-1477-8

**Published:** 2017-12-08

**Authors:** Michelle Hall, Rana S. Hinman, Martin van der Esch, Marike van der Leeden, Jessica Kasza, Tim V. Wrigley, Ben R. Metcalf, Fiona Dobson, Kim L. Bennell

**Affiliations:** 1 Centre for Health, Exercise and Sports Medicine, Department of Physiotherapy, School of Health Sciences, The University of Melbourne, Melbourne, VIC 3010 Australia; 2 Amsterdam Rehabilitation Research Center Reade, Amsterdam, The Netherlands; 3 Department of Epidemiology and Preventive Medicine, Monash University, Melbourne, VIC Australia; 4 Department of Physiotherapy, School of Health Sciences, The University of Melbourne, Melbourne, VIC Australia

**Keywords:** Exercise, Knee muscle, Physical function, Knee osteoarthritis

## Abstract

**Background:**

Clinical guidelines recommend knee muscle strengthening exercises to improve physical function. However, the amount of knee muscle strength increase needed for clinically relevant improvements in physical function is unclear. Understanding how much increase in knee muscle strength is associated with improved physical function could assist clinicians in providing appropriate strength gain targets for their patients in order to optimise outcomes from exercise. The aim of this study was to investigate whether an increase in knee muscle strength is associated with improved self-reported physical function following exercise; and whether the relationship differs according to physical function status at baseline.

**Methods:**

Data from 100 participants with medial knee osteoarthritis enrolled in a 12-week randomised controlled trial comparing neuromuscular exercise to quadriceps strengthening exercise were pooled. Participants were categorised as having mild, moderate or severe physical dysfunction at baseline using the Western Ontario and McMaster Universities Osteoarthritis Index (WOMAC). Associations between 12-week changes in physical function (dependent variable) and peak isometric knee extensor and flexor strength (independent variables) were evaluated with and without accounting for baseline physical function status and covariates using linear regression models.

**Results:**

In covariate-adjusted models without accounting for baseline physical function, every 1-unit (Nm/kg) increase in knee extensor strength was associated with physical function improvement of 17 WOMAC units (95% confidence interval (CI) −29 to −5). When accounting for baseline severity of physical function, every 1-unit increase in knee extensor strength was associated with physical function improvement of 24 WOMAC units (95% CI −42 to −7) in participants with severe physical dysfunction. There were no associations between change in strength and change in physical function in participants with mild or moderate physical dysfunction at baseline. The association between change in knee flexor strength and change in physical function was not significant, irrespective of baseline function status.

**Conclusions:**

In patients with severe physical dysfunction, an increase in knee extensor strength and improved physical function were associated.

**Trial registration:**

ANZCTR 12610000660088. Registered 12 August 2010.

**Electronic supplementary material:**

The online version of this article (10.1186/s13075-017-1477-8) contains supplementary material, which is available to authorized users.

## Background

People with knee osteoarthritis (OA) often have difficulty performing activities of daily living and physical dysfunction is a key driver for total knee arthroplasty eligibility [1]. Knee muscle weakness is a typical feature of knee OA [2] and is associated with physical dysfunction in people with the disease [3]. Clinical guidelines recommend knee muscle strengthening exercises to improve physical function [4, 5]. However, the amount of knee muscle strength increase needed for clinically relevant improvements in physical function is unclear. Understanding how much increase in knee muscle strength is associated with improved physical function could assist clinicians in providing appropriate strength gain targets for their patients in order to optimise outcomes from exercise.

In people with knee OA, deficits in knee extensor and flexor strength, relative to body mass, range between 20 and 40% compared to individuals without knee OA [6–10]. Evidence from observational [11, 12] and pre–post exercise [13, 14] studies supports an association between change in knee muscle strength and change in self-reported physical function in people with [11–14] or at risk for [11] knee OA. Although these study designs preclude causal inferences, these studies provide some insight into the magnitude of increase in knee muscle strength potentially associated with physical function improvement. However, interpretation is limited by the assumption that the relationship between change in strength and function is consistent across all patients, irrespective of baseline dysfunction. Previous research has determined that the magnitude of the minimal clinically important improvement (MCII) in physical function in people with knee OA depends on baseline physical function status [15]. Specifically, patients with less difficulty with physical function require less improvement in physical function to have a clinical meaningful improvement compared to patients with more severe physical dysfunction [15]. Therefore, it is possible that relationships between changes in knee muscle strength and physical function may be influenced by baseline physical function status. However, this has not been evaluated to date.

In a randomised controlled trial (RCT), we compared outcomes at 12 weeks from two exercise programmes (weight-bearing neuromuscular exercise versus non-weight-bearing quadriceps strengthening) in people with medial tibiofemoral knee OA and varus alignment [16]. Comparable between-group improvements in self-reported physical function and knee muscle strength over the 12-week study period were found with both exercise programmes [16]. Using pooled data from this RCT, the purpose of this exploratory study was to evaluate the association between change in knee muscle strength and change in self-reported physical function in patients following 12 weeks of exercise and to evaluate magnitudes of association according to baseline severity in physical dysfunction.

## Methods

### Participants

Data from people who participated in a RCT were used [16]. Between July 2010 and July 2011, 100 people aged > 50 years with medial tibiofemoral compartment knee OA and varus malalignment were recruited from the community. Individuals were eligible if they had: radiographic medial tibiofemoral knee OA (defined as Kellgren and Lawrence grade 2 or greater [17]) with greater medial tibiofemoral joint space narrowing compared to lateral tibiofemoral joint narrowing [18], and medial compartment osteophyte severity greater than or equal to lateral compartment osteophyte severity [18]; static varus alignment on radiograph (defined as a mechanical axis angle of < 181° for females and < 183° for males) [19]; and average knee pain over the past week of ≥ 25 on a 100-mm visual analogue scale (VAS). Exclusion criteria included: knee surgery or an intraarticular corticosteroid injection within the last 6 months; oral corticosteroid use current or within the past 4 weeks; systemic arthritic conditions; prior joint replacement (hip or knee) or tibial osteotomy surgery; other non-pharmacologic treatment within the past 6 months; or body mass index > 36 kg/m^2^. The most symptomatic knee was deemed the study knee in cases of bilaterally eligible cases. Ethical approval was obtained from the University of Melbourne Human Ethics Committee and all participants provided written informed consent.

### Interventions

Participants were randomised to one of two 12-week home-based exercise programmes (either neuromuscular exercise or traditional quadriceps strengthening exercises) [16]. Participants were supervised by a physiotherapist for 14 sessions and were instructed to exercise a minimum of 4 days per week [16]. Further detail on the exercise programmes and the trial protocol has been published previously [20].

### Dependent variable (outcome)

Self-reported physical function was assessed using the WOMAC physical function subscale with knee-related questions on a scale from 0 (‘none’) to 4 (‘extreme’). The total score was normalised to a 0–100 score, where higher scores indicate extreme difficulty [21]. The WOMAC has been demonstrated previously as reliable, valid and responsive [21]. Improvements of 5.3, 11.8 and 20.4 points on the WOMAC physical function scale have been associated previously with a MCII in people with knee OA with mild, moderate and severe physical dysfunction respectively [15].

### Independent variables

A KinCom 125-AP isokinetic dynamometer (Chattecx, Chattanooga, TN, USA) was used to assess maximal isometric knee extensor and knee flexor strength at 60° knee flexion. Following submaximal efforts, participants performed three maximal trials while receiving strong verbal encouragement to “push/pull as hard as you can” for the knee extensor and knee flexor muscles respectively. The distance from the ankle cuff to the rotation axis of the dynamometer was used as the lever arm length. The peak force (Newtons) from three maximal contractions (gravity compensated) was multiplied by the lever arm length (m) and divided by body mass (kg). Muscle strength was normalised to body mass (kg) as a large proportion of the items on the WOMAC physical function subscale involve weight-bearing activities [21].

### Other measures

Average overall knee pain during the past week was assessed using a 100-mm VAS with endpoints of “no pain” and “worst pain possible” [22]. Disease severity was assessed using the Kellgren and Lawrence grading scale [17]. Participants were graded as either grade 2 (definitive osteophytes with possible narrowing of joint space), grade 3 (moderate multiple osteophytes, definite narrowing of joint space and some sclerosis and possible deformity of bone ends) or grade 4 (large osteophytes, marked narrowing of joint space, severe sclerosis and definite deformity of bone ends). Anatomic knee alignment was assessed from radiographs according to previously described methods [23].

### Statistical analysis

Analyses were performed using Stata software, version 13.1 (Statacorp, College Station, TX, USA) and significance was set at *p* < 0.05. Data from both exercise groups were pooled because no significant interactions between exercise group (i.e. neuromuscular exercise and quadriceps strengthening groups) and strength were observed (Additional file 1: Table S1). One-way analyses of variance and Pearson chi-squared tests were used to compare baseline participant characteristics across the three levels of physical dysfunction severity for continuous and categorical data respectively. For descriptive purposes, paired *t* tests were used to determine change in knee muscle strength and symptoms for the cohort and each category of each physical dysfunction.

Missing follow-up data were imputed (*n* = 8 self-reported physical function; *n* = 20 knee muscle strength) using chained equations with predictive mean matching and a neighbourhood size of three. The multiple imputation model included change in knee extensor strength, change in knee flexor strength, change in VAS pain, change in WOMAC pain, change in WOMAC physical function, sex, age, BMI and the baseline score of each measures of knee strength (extensor and flexor) and symptoms (pain and function). Estimates from 20 imputed data sets were combined using Rubin’s rules. Sensitivity analyses were performed using complete case analyses (*n* = 80) (Additional file 2: Table S2). Using previously described cut-off points in WOMAC physical function (0–100) [15], scores ≤ 35.5 were classified as mild physical dysfunction, scores ranging between 35.4 and 51.5 were classified as moderate physical dysfunction and scores > 51.5 were classified as severe physical dysfunction.

Separate linear regression models for knee extensor strength and knee flexor strength were used to estimate the association between change in strength (independent variable) and change in physical function (dependent variable), initially without considering baseline physical dysfunction. In models that considered changes in strength by baseline physical dysfunction severity, baseline physical dysfunction severity and an interaction term between the change in muscle strength and baseline physical function severity (e.g. 12-week change in knee extensor strength × baseline physical function severity) were also included. Regression models were unadjusted, as well as adjusted for age, sex, exercise group, baseline strength and change in pain (VAS). For each measure of strength, the interaction term (change in knee extensor strength × baseline physical dysfunction severity) was interrogated to yield a coefficient for each level of physical dysfunction. Residuals of each linear model were inspected using plots of for normality (normal quantile–quantile plots) and constant variance (scatter plots).

## Results

Descriptive statistics for the cohort, and according to baseline physical dysfunction severity, are presented in Table 1. In general, the cohort was middle-aged and overweight, with both sexes represented equally. Further to this, participants were relatively comparable when categorised by severity of baseline physical function (Table 1) with few exceptions. Pain assessed using the VAS was significantly greater in participants with severe physical dysfunction compared to participants with mild and moderate dysfunction (Table 1). Knee extensor strength significantly increased for participants with mild, moderate and severe physical dysfunction at baseline (Table 2) by 5%, 8% and 13% respectively. Knee flexor strength did not increase significantly according to baseline physical dysfunction severity (Table 2). Symptoms significantly improved for participants with mild, moderate and severe physical dysfunction (Table 2).

**Table 1:** Baseline participant characteristics in the entire cohort and when categorised based on baseline physical dysfunction severity

	Total cohort (*n* = 100)	Baseline physical dysfunction severity		
		Mild (*n* = 38)	Moderate (*n* = 43)	Severe (*n* = 19)
Age (years)	62.4 ± 7.3	62.4 ± 6.8	62.2 ± 7.8	63.0 ± 7.6
Women, *n* (%)	52 (52%)	20 (53%)	22 (51%)	10 (53%)
Height (m)	1.67 ± 0.10	1.67 ± 0.10	1.68 ± 0.09	1.65 ± 0.11
Mass (kg)	82.7 ± 14.3	82.6 ± 15.2	84.0 ± 14.7	79.8 ± 11.5
Body mass index (kg/m^2^)	29.64 ± 4.08	29.67 ± 4.29	29.69 ± 4.08	29.50 ± 3.85
Knee alignment^a^ (degrees)	176.8 ± 3.5	176.6 ± 3.7	176.5 ± 3.4	177.9 ± 3.0
Neuromuscular exercise: quadriceps strengthening group	50:50	20:18	24:19	6:13
Bilateral osteoarthritis, yes:no	47:53	15:23	23:20	9:10
Radiographic disease severity^b^				
Grade 2	22 (22%)	7 (18%)	11 (26%)	4 (21%)
Grade 3	43 (43%)	16 (42%)	17 (40%)	10 (53%)
Grade 4	35 (35%)	15 (39%)	15 (35%)	5 (26%)
VAS average knee pain in the past week, 0–100 mm^c^	54.1 ± 15.0	52.8 ± 15.1	51.3 ± 12.5	62.8 ± 17.7^d,e^
WOMAC physical function, 0–100^c^	39.8 ± 14.1	26.2 ± 8.5	43.2 ± 4.5^d^	59.3 ± 8.2^d,e^
Knee extensor strength (Nm/kg)	1.45 ± 0.45	1.49 ± 0.48	1.50 ± 0.43	1.27 ± 0.39
Knee flexor strength (Nm/kg)	0.69 ± 0.22	0.69 ± 0.19	0.70 ± 0.24	0.64 ± 0.23

*WOMAC* Western Ontario and McMaster Universities Osteoarthritis Index, *VAS* visual analogue scale

^a^Anatomic alignment, where neutral alignment is 181° for females and 183° for males. Varus is < 181° for females and < 183 ° for males [19]

^b^Kellgren and Lawrence grade

^c^Higher scores indicates greater pain/dysfunction

^d^Significantly different to mild physical dysfunction (*p* < 0.05)

^e^Significantly different to moderate physical dysfunction (*p* < 0.05)

**Table 2:** Change (follow-up minus baseline) in knee muscle strength

	Baseline physical dysfunction severity			
	Total group	Mild	Moderate	Severe
Knee extensor strength (Nm/kg)	0.12 (0.07 to 0.17)^*^	0.08 (0.02 to 0.15)^*^	0.12 (0.06 to 0.19)^*^	0.17 (0.00 to 0.33)^*^
Knee flexor strength (Nm/kg)	0.05 (0.02 to 0.07)^*^	0.03 (−0.02 to 0.07)	0.05 (0.01 to 0.09)	0.08 (0.01 to 0.15)
WOMAC physical function	−11.07 (−13.59 to −8.54)^*^	−3.92 (−7.26 to −0.57)^*^	−14.69 (−18.23 to −11.14)^*^	−17.18 (−23.04 to −11.32)^*^
WOMAC pain	−10.40 (−13.10 to − 7.70)^*^	−6.73 (−11.41 to −2.05)^*^	−10.91 (−14.83 to −6.98)^*^	−16.59 (−21.76 to −11.44)^*^
VAS pain	−20.80 (−25.33 to −16.27)^*^	−22.81 (−30.23 to −15.39)^*^	−18.68 (−25.16 to −12.21)^*^	−21.58 (−33.74 to −9.42)^*^

Data presented as mean (95% confidence interval)

*WOMAC* Western Ontario and McMaster Universities Osteoarthritis Index, *VAS* visual analogue scale

*p < 0.05

### Knee extensor strength

Without accounting for baseline physical function status, change in knee extensor muscle strength along with all covariates considered in this study explained 10% of the variation in the change in physical function. A 1-unit increase (Nm/kg) in peak knee extensor strength was associated with a 17-unit improvement (95% CI −29 to −5) in WOMAC physical function. In another model, accounting for baseline physical function status and an interaction with change in knee extensor strength along with all covariates, 33% of the variation in the change in physical function was explained. In this model, a 1-unit increase (Nm/kg) in peak isometric knee extensor strength corresponded to a 24-unit improvement (95% CI −42 to −7) in WOMAC physical function score in participants with severe baseline dysfunction. Notably, the association between change in knee extensor strength and change in physical function was not significant for participants with mild and moderate baseline physical dysfunction (Table 3). Sensitivity analyses using only complete cases (*n* = 80) demonstrated similar results for change in knee extensor strength as the independent variable (Additional file 2: Table S2).

**Table 3:** Linear relationships between 12-week change in strength-related measures (independent variable) and 12-week change in WOMAC function (dependent variable) for the entire cohort and according to physical dysfunction severity at baseline using imputed data

		Univariable analysis			Multivariable analysis^a^			Multivariable analysis^b^			Multivariable analysis^c^		
		Regression coefficient (95% CI)	*p* value	Adj. *R* ^2^	Regression coefficient (95% CI)	*p* value	Adj. *R* ^2^	Regression coefficient (95% CI)	*p* value	Adj. *R* ^2^	Regression coefficient (95% CI)	*p* value	Adj. *R* ^2^
Entire cohort													
Δ Knee extensor strength (Nm/kg)		−17.3 (−29.5 to −5.2)	0.01	0.09	−17.7 (−30.0 to −5.3)	0.01	0.08	−17.9 (−30.5 to −5.3)	0.01	0.06	−17.2 (−29.4 to −4.9)	0.01	0.10
Δ Knee flexor strength (Nm/kg)		−19.6 (−44.4 to 5.3)	0.12	0.03	−19.0 (−44.0 to 6.0)	0.13	0.05	−19.3 (−44.6 to 6.0)	0.13	0.00	−22.0 (−46.4 to 2.5)	0.08	0.06
According to baseline physical dysfunction													
Δ Knee extensor strength (Nm/kg)				0.27^d^			0.27^d^			0.26^d^			0.33^d^
	Mild	−2.7 (−26.1 to 20.8)	0.82		−1.7 (−25.9 to 22.5)	0.89		−2.2 (−26.7 to 22.2)	0.86		4.8 (−20.2 to 29.8)	0.70	
	Moderate	−13.4 (−32.6 to 5.9)	0.17		−13.8 (−32.7 to 5.2)	0.15		−13.4 (−33.3 to 6.4)	0.18		−16.9 (−35.7 to 1.9)	0.08	
	Severe	−24.0 (−42.2 to −5.7)	0.01		−24.8 (−43.1 to −6.4)	0.01		−24.9 (−43.1 to −6.5)	0.01		−24.2 (−41.7 to −6.7)	0.01	
Δ Knee flexor strength (Nm/kg)				0.22^d^			0.21^d^			0.20^d^			0.27^d^
	Mild	0.01 (−34.8 to 35.0)	0.99		−2.8 (−33.2 to 38.8)	0.88		2.20 (−34.4 to 38.8)	0.90		−7.3 (−44.3 to 29.7)	0.69	
	Moderate	−13.9 (−45.0 to 17.2)	0.38		−13.8 (−44.5 to 16.8)	0.37		−14.21 (−45.2 to 16.8)	0.36		−10.2 (−39.8 to 19.3)	0.49	
	Severe	−42.0 (−99.6 to 15.6)	0.15		−42.7 (−101.3 to 16.0)	0.15		−42.00 (−100.8 to 16.8)	0.16		−47.6 (−100.7 to 5.6)	0.08	

*Adj.* adjusted, *CI* confidence interval, *WOMAC* Western Ontario and McMaster Universities Osteoarthritis Index

^a^Adjusted for sex, age

^b^Adjusted for sex, age, exercise group, baseline strength

^c^Adjusted for sex, age, exercise group, baseline strength, change in pain (visual analogue scale)

^d^Adjusted *R* ^2^ value for the entire model including all terms

### Knee flexor strength

Without accounting for baseline physical function status, increased knee flexor muscle strength along with all covariates considered in this study explained 6% of the variation in the change in physical function. However, the association between change in knee flexor strength and change in physical function was not statistically significant in the model unadjusted for covariates or in the models adjusted for any or all covariates (Table 3). When accounting for baseline physical function status and an interaction with change in knee flexor strength along with all covariates, 27% of the variation in the change in physical function was explained. In this model, the association between change in knee flexor strength and change in physical function was not significant, irrespective of baseline physical function status. Sensitivity analyses using only complete cases (*n* = 80) demonstrated a statistically significant association between increased knee flexor strength and improvement in physical function without accounting for baseline physical function status. In the sensitivity analyses accounting for baseline physical function status and an interaction with change in knee flexor strength, there was a significant association between increased knee flexor strength and improved physical function in those with severe physical dysfunction only (Additional file 2: Table S2).

## Discussion

We observed a statistically significant association between increased knee extensor strength and improved self-reported physical function in people with knee OA who underwent exercise therapy. However, when investigating the cohort according to baseline severity of physical dysfunction, there was limited evidence of associations between change in knee muscle strength and change in physical function for participants with mild and moderate physical dysfunction at baseline. Conversely, for participants with severe physical dysfunction at baseline, there was a significant association between increased knee extensor strength and improved self-reported physical function. Taken together, the findings of this study provide preliminary evidence to suggest that the relationship between increased knee extensor strength and self-reported physical function improvement may depend on baseline levels of physical dysfunction.

Understanding the association between change in muscle strength and improved physical function following exercise is important. Exercise is a cornerstone treatment for knee OA [4, 5] and self-reported physical function using the WOMAC is recommended as an end-point in OA clinical trials [22]. Similar to previous research [13, 14, 24] we observed a statistically significant association between increased knee extensor strength and improvement in physical function without accounting for physical dysfunction severity at baseline. Based on the regression equation, the 0.12 Nm/kg (8%) average increase in knee extensor strength was associated with a 2.05-unit improvement (95% CI −3.5 to −0.6) in WOMAC physical function. In the context of strength gains in people with knee OA, the strength gains in the current study are lower than mean increases of 17% (range 10.5% decrease to 49.5% increase) in response to resistance training in people with knee OA reported in a systematic review [25]. Notably, the relationship between self-reported physical function and variables of knee muscle strength, age, sex, exercise group, baseline strength and change in pain (VAS) explained 6–10% of the variation in self-reported physical function. In contrast to a 38-week longitudinal study where participants performed exercise [14], we did not observe an association between increases in knee flexor strength and improved self-reported function on the WOMAC despite the significant improvement in knee flexor strength we reported previously [16]. Several between-study differences such as strength measurement protocols, study duration and participant characteristics may account for the inconsistent findings. Further to this, the exercise interventions used in the current study did not specifically target knee flexor strength. Thus, changes in knee flexor strength were relatively small (Table 2), which may have contributed to a lack of statistical power to detect a statically significant association. Nonetheless, we observed potential evidence to support an association between change in knee flexor strength and improvement in physical function (48.6-unit improvement (95% CI −100.7 to 5.6) in WOMAC physical function), and hence we are hesitant to disregard the potential for a relationship. There was a statistically significant association between increased knee flexor strength and physical function improvement when analysing complete cases (Additional file 2: Table S2).

Our study extends existing knowledge by describing the association between knee muscle strength and self-reported function according to three baseline categories of physical dysfunction. Interestingly, the amount of variation explained by the relationship between physical function and knee extensor strength, age, sex, exercise group, baseline strength and change in pain (VAS) increased from 10 to 33% for the regression models when the baseline level of physical dysfunction was considered. In participants with severe physical dysfunction at baseline, increased knee extensor strength significantly contributed to improved physical function. Specifically, for participants with severe physical dysfunction at baseline, the 0.17 Nm/kg (13%) average increase in knee extensor strength was associated with a 4.1-unit improvement (CI 95% −7.1 to −1.1) in WOMAC physical function. The MCII for WOMAC physical function in knee OA patients with severe physical dysfunction has been estimated to be 20.4 units [15]. Therefore, based on the regression equation, a much larger gain in knee extensor strength of 0.85 Nm/kg (67%) is associated with a MCII in physical function for patients with severe physical dysfunction at baseline. Interestingly, only one of the 19 participants with severe physical dysfunction at baseline increased knee extensor strength ≥ 0.85 Nm/kg over the 12 weeks. Overall, it appears that an increase in knee extensor strength associated with a clinically relevant improvement in physical function is potentially achievable for few patients. Our findings suggest that resistance programmes should aim for greater increases in knee extensor strength, as this may yield greater self-reported physical function improvement for people with severe physical dysfunction at baseline. It is important to acknowledge that a large proportion of the variation in physical function remains unexplained (67%) by a linear relationship between physical function and knee muscle strength together with covariates. Future research is required to validate whether these estimates of knee extensor strength increases yield a MCII in physical function for knee OA patients with severe physical dysfunction in response to exercise.

In contrast to participants with severe physical dysfunction, changes in knee muscle strength were not associated with changes in physical function for participants with mild or moderate physical dysfunction at baseline. Reasons for differences in the associations depending on severity of baseline physical dysfunction are unclear. Increases in knee muscle strength were not statistically different across the levels of physical dysfunction, when accounting for baseline level of knee muscle strength (data not shown). Thus, factors other than increased maximal isometric knee extension/flexion strength appear to contribute to a clinically relevant improvement in physical function [15] following exercise for many participants. Various clinical and psychological factors predict deterioration in self-reported physical function [26] in people with knee OA. However, little is known regarding the association of these factors to improved physical function following exercise. In people with OA, treatment expectation has been shown to moderate the effectiveness of cognitive behavioural therapy [27] and there is some evidence to suggest that treatment expectations are related to clinical outcomes from exercise and acupuncture [28]. A recent study suggests that knee OA patients with higher treatment expectancy for exercise have greater self-efficacy and fewer depressive symptoms compared to patients with lower treatment expectancy [29]. The unexplained variation in self-reported physical function change observed in the current study may in part be accounted for by treatment expectation, self-efficacy and depressive symptoms. Future research should consider whether improvement in these factors, among others [30], differs according to physical dysfunction.

From a clinical perspective, our data suggest that either exercise programme used in this study is beneficial to improve physical function, irrespective of baseline physical function. Post-hoc analyses confirmed that improvement in physical function was no different between exercise groups according to the baseline level of physical dysfunction (interaction *p* = 0.39). Hence, this study does not question the efficacy of knee strengthening interventions to improve  physical function. Instead, this study generates the hypotheses that physical function improvement is associated with factors other than increased knee extensor strength for patients with mild or moderate physical dysfunction at baseline, and that gains in knee extensor strength only partially account for improved physical function in patients with severe physical dysfunction.

Evidently, a greater understanding of how physical function improves in patients with knee OA following strengthening exercise is needed so that exercise prescription can be improved to optimise treatments.

Strengths of our study include a relatively large cohort with excellent adherence to the exercise interventions (median percentage of home exercises completed was 82% by the neuromuscular exercise group and 91% by the quadriceps strengthening exercise group) [16]. Limitations of our study also warrant consideration. First, as exploratory analyses, our findings are preliminary and require validation with larger samples. Our small sample size limits the accuracy of estimates as reflected by the wide confidence intervals. Second, although our data suggest that increased maximal isometric knee extensor strength is a potential mechanism underpinning improvement in physical function due to exercise for those with severe physical dysfunction at baseline, effects of exercise can only be determined by analysing effect modification by group (exercise versus no exercise). Third, the cut-off points used to categorise the severity of physical dysfunction were based on knee OA literature demonstrating that a MCII in physical function in response to non-steroidal anti-inflammatory drugs was dependent on the severity of physical dysfunction at baseline [15]. Thus, the cut-off points used to categorise physical function severity may not necessarily apply to exercise treatments. However, findings from regression analyses remained unchanged when using baseline physical function cut-off points based on tertiles (Additional file 3: Table S3). Fourth, our results can only be generalised to the two 12-week exercise programmes evaluated in the original trial. Similarly, due to the patient selection criteria of the original clinical trial, our findings are only generalisable to patients with medial knee OA and varus malalignment who report moderate levels of knee pain. Hence our findings may not be applicable to all patients with knee OA. Also, participants in the current study volunteered to be in an exercise trial, and thus may be more motivated to exercise than the average patient with knee OA. Lastly, our results cannot be generalised to self-reported measures of physical function beyond the WOMAC or to objective measures of physical function.

## Conclusions

Overall, we found preliminary evidence to suggest that the association between change in knee muscle strength and improvement in self-reported physical function following 12 weeks of exercise therapy differs according to baseline difficulty with physical function. This may facilitate future research to optimise treatment effects of exercise. Further research is required to investigate factors that associate with improvement in physical function for knee OA patients, particularly those with mild to moderate physical dysfunction.

## Additional files

Additional file 1: Table S1. Linear relationships between measure of peak isokinetic knee muscle strength (independent variable) and WOMAC physical function 100 scale (dependent variable), including interaction between ‘exercise program’ and ‘measure of strength’. (DOCX 20 kb)

Additional file 2: Table S2. Linear relationships between change in strength-related measures (independent variable) and change on WOMAC function (dependent variable) according to physical dysfunction severity at baseline (complete cases n =80). (DOCX 21 kb)

Additional file 3: Table S3. Linear relationships between 12-week change in strength-related measures (independent variable) and 12-week change on WOMAC physical function (dependent variable) according to tertiles of physical dysfunction severity at baseline. (DOCX 26 kb)

## Abbreviations

MCII: Minimal clinical important improvement
OA: Osteoarthritis
RCT: Randomised controlled trial
VAS: Visual analogue scale
WOMAC: Western Ontario and McMaster Universities Osteoarthritis Index
