# Supplementary material for: Is the relationship between increased knee muscle strength and improved physical function following exercise dependent on baseline physical function status?
Source: Arthritis Res Ther. 2017 Dec 8;19:271. doi: 10.1186/s13075-017-1477-8 (PMC5721363; doi:10.1186/s13075-017-1477-8)
Supplement: Supplementary file 1 — Linear relationships between measure of peak isokinetic knee muscle strength (independent variable) and WOMAC physical function 100 scale (dependent variable), including interaction between ‘exercise program’ and ‘measure of strength’. (DOCX 20 kb) [file 13075_2017_1477_MOESM1_ESM.docx]

| **Table S1.** Linear relationships between measure of peak isokinetic knee muscle strength (independent variable) and WOMAC physical function 100 scale (dependent variable), including interaction between ‘exercise program’ and ‘measure of strength’ | | | | | | | |
| --- | --- | --- | --- | --- | --- | --- | --- |
|  |  |  | Simple Regression | Interaction  p - value |  | Multiple Regression^1^ | Interaction p - value |
| **Complete cases (n = 80)** |  |  |  |  |  |  |  |
| Knee quadriceps strength (Nm/kg) |  | Exercise Program 1 | -0.13 (-9.9 to 9.7) | 0.99 |  | -17.36 (-37.0 to 2.30) | 0.87 |
|  |  | Exercise Program 2 | -0.03 (-12.1 to -12.0) |  |  | -15.33 (-29.4 to -1.3) |  |
|  |  |  |  |  |  |  |  |
| Knee flexion strength (Nm/kg) |  | Exercise Program 1 | -34.65 (-63.6 to -5.7) | 0.36 |  | -34.42 (-64.7 to -4.2) | 0.39 |
|  |  | Exercise Program 2 | -16.15 (-43.8 to 11.5) |  |  | -16.54 (-44.6 to 11.5) |  |
|  |  |  |  |  |  |  |  |
| **Imputed data (n = 100)** |  |  |  |  |  |  |  |
| Knee quadriceps strength (Nm/kg) |  | Exercise Program 1 | -20.52 (-40.1 to -1.0) | 0.67 |  | -20.79 (-40.0 to -1.6) | 0.61 |
|  |  | Exercise Program 2 | -15.24 (-30.4 to -0.1) |  |  | -14.59 (-29.8 to 0.6) |  |
|  |  |  |  |  |  |  |  |
| Knee flexion strength (Nm/kg) |  | Exercise Program 1 | -25.04 (-60.5 to 10.5) | 0.63 |  | -29.27 (-64.5 to 6.0) | 0.52 |
|  |  | Exercise Program 2 | -13.95 (-45.9 to 18.0) |  |  | -14.65 (-46.0 to 16.7) |  |
|  |  |  |  |  |  |  |  |
| ^1^ adjusted for gender, age, exercise group, baseline strength, change in pain (VAS)  Exercise program 1: Neuromuscular exercise  Exercise program 2: Quadriceps strengthening exercise | | | | | | | |
