# Supplementary material for: Is the relationship between increased knee muscle strength and improved physical function following exercise dependent on baseline physical function status?
Source: Arthritis Res Ther. 2017 Dec 8;19:271. doi: 10.1186/s13075-017-1477-8 (PMC5721363; doi:10.1186/s13075-017-1477-8)
Supplement: Supplementary file 2 — Linear relationships between change in strength-related measures (independent variable) and change on WOMAC function (dependent variable) according to physical dysfunction severity at baseline (complete cases n =80). (DOCX 21 kb) [file 13075_2017_1477_MOESM2_ESM.docx]

| **Table S2** Linear relationships between change in strength-related measures (independent variable) and change on WOMAC function (dependent variable) according to physical dysfunction severity at baseline (complete cases n =80) | | | | | | | | | | | | | |
| --- | --- | --- | --- | --- | --- | --- | --- | --- | --- | --- | --- | --- | --- |
|  |  | **Univariable analysis** | **Slope** | **Adj R^2^** | **Multivariable analysis^1^** | **Slope** | **Adj R^2^** | **Multivariable analysis^2^** | **Slope** | **Adj R^2^** | **Multivariable analysis^3^** | **Slope** | **Adj R^2^** |
|  |  | **Regression coefficient**  **(95%CI)** | **p Value** |  | **Regression coefficient**  **(95%CI)** | **p Value** |  | **Regression coefficient**  **(95%CI)** | **p Value** |  | **Regression coefficient**  **(95%CI)** | **p Value** |  |
|  |  |  |  |  |  |  |  |  |  |  |  |  |  |
| Δ Knee quadriceps strength (Nm/kg) |  | -16.1 (-27.0 to -5.2) | <0.01 | 0.09 | -16.6 (-27.6 to -5.5) | <0.01 | 0.09 | -16.0 (-27.3 to -4.6) | <0.01 | 0.08 | -16.0 (-27.4 to -4.6) | 0.01 | 0.07 |
| Δ Knee hamstring strength (Nm/kg) |  | -24.5 (-44.4 to -4.6) | 0.02 | 0.06 | -23.3 (-43.4 to -3.2) | 0.02 | 0.05 | -23.6 (-44.0 to -3.3) | 0.02 | 0.04 | -24.8 (-45.3 to -4.3) | 0.02 | 0.04 |
|  | According to baseline physical dysfunction |  |  |  |  |  |  |  |  |  |  |  |  |
| Δ Knee quadriceps strength (Nm/kg) |  |  |  | 0.28 |  |  | 0.29 |  |  | 0.35 |  |  | 0.40 |
|  | Mild | -1.3 (-20.6 to 18.1) | 0.90 |  | -0.4 (-20.1 to 19.3) | 0.97 |  | 0.6 (-19.6 to 20.9) | 0.95 |  | 5.7 (-14.4 to 25.8) | 0.57 |  |
|  | Moderate | -13.3 (-29.3 to 2.8) | 0.11 |  | -14.2 (-30.3 to 1.8) | 0.08 |  | -13.4 (-30.4 to 3.5) | 0.12 |  | -16.2 (-32.8 to 0.4) | 0.06 |  |
|  | Severe | -23.7 (-39.5 to -7.8) | <0.01 |  | -24.7 (-40.5 to -8.8) | <0.01 |  | -24.8 (-40.8 to -8.7) | <0.01 |  | -25.3 (-40.9 to -9.7) | <0.01 |  |
| Δ Knee hamstring strength (Nm/kg) |  |  |  | 0.29 |  |  | 0.30 |  |  | 0.22 |  |  | 0.25 |
|  | Mild | -7.0 (-37.6 to 23.6) | 0.65 |  | -2.9 (-34.1 to 28.4) | 0.856 |  | -4.2 (-36.3 to 28.0) | 0.80 |  | -11.1 (-43.3 to 21.1) | 0.49 |  |
|  | Moderate | -14.6 (-40.9 to 11.7) | 0.27 |  | -15.0 (-41.4 to 11.3) | 0.260 |  | -14.6 (-41.4 to 12.2) | 0.28 |  | -11.7 (-38.1 to 14.7) | 0.38 |  |
|  | Severe | -54.4 (-99.8 to -9.0) | 0.02 |  | -54.6 (-100.1 to -9.1) | 0.019 |  | -54.3 (-100.5 to -8.0) | 0.02 |  | -57.6 (-102.8 to -12.1) | 0.01 |  |
| ^1^ adjusted for gender, age  ^2^ adjusted for gender, age, exercise group, baseline strength  ^3^ adjusted for gender, age, exercise group, baseline strength, change in pain (VAS) | | | | | | | | | | | | | |
