# Supplementary material for: Is the relationship between increased knee muscle strength and improved physical function following exercise dependent on baseline physical function status?
Source: Arthritis Res Ther. 2017 Dec 8;19:271. doi: 10.1186/s13075-017-1477-8 (PMC5721363; doi:10.1186/s13075-017-1477-8)
Supplement: Supplementary file 3 — Linear relationships between 12-week change in strength-related measures (independent variable) and 12-week change on WOMAC physical function (dependent variable) according to tertiles of physical dysfunction severity at baseline. (DOCX 26 kb) [file 13075_2017_1477_MOESM3_ESM.docx]

| **Table S3** Linear relationships between 12-week change in strength-related measures (independent variable) and 12-week change on WOMAC physical function (dependent variable) according to tertiles of physical dysfunction severity at baseline | | | | | | | | | | | | | |
| --- | --- | --- | --- | --- | --- | --- | --- | --- | --- | --- | --- | --- | --- |
|  |  | **Univariable analysis** | **Slope** | **Adj R^2^** | **Multivariable analysis^1^** | **Slope** | **Adj R^2^** | **Multivariable analysis^2^** | **Slope** | **Adj R^2^** | **Multivariable analysis^3^** | **Slope** | **Adj R^2^** |
|  |  | **Regression coefficient**  **(95%CI)** | **p Value** |  | **Regression coefficient**  **(95%CI)** | **p Value** |  | **Regression coefficient**  **(95%CI)** | **p Value** |  | **Regression coefficient**  **(95%CI)** | **p Value** |  |
|  | According to baseline physical dysfunction |  |  |  |  |  |  |  |  |  |  |  |  |
| **Complete cases (n=80)** |  |  |  |  |  |  |  |  |  |  |  |  |  |
| Δ Knee quadriceps strength (Nm/kg) |  |  |  | 0.27 |  |  | 0.29 |  |  | 0.27 |  |  | 0.32 |
|  | Mild | 0.9 (-19.23 to 20.9) | 0.93 |  | 1.6 (-18.6 to 21.9) | 0.87 |  | 2.80 (-18.2 to 23.8) | 0.79 |  | 8.1 (-12.6 to 28.8) | 0.44 |  |
|  | Moderate | -14.9 (-33.84 to 4.1) | 0.12 |  | -16.8 (-35.7 to 2.1) | 0.08 |  | -15.6 (-35.3 to 4.0) | 0.12 |  | -17.3 (-36.3 to 1.8) | 0.08 |  |
|  | Severe | -21.3 (-35.40 to -7.2) | <0.01 |  | -21.3 (-35.2 to -7.3) | <0.01 |  | -21.2 (-35.3 to -7.1) | <0.01 |  | -22.8 (-36.5 to -9.1) | <0.01 |  |
| Δ Knee hamstring strength (Nm/kg) |  |  |  | 0.22 |  |  | 0.22 |  |  | 0.21 |  |  | 0.24 |
|  | Mild | -5.2 (-36.53 to 26.2) | 0.74 |  | -0.5 (-32.7 to 31.3) | 0.98 |  | -2.4 (-34.9 to 30.0) | 0.88 |  | -9.3 (-41.9 to 23.3) | 0.57 |  |
|  | Moderate | -13.2 (-43.80 to 17.4) | 0.39 |  | -12.3 (-42.8 to 18.1) | 0.42 |  | -10.5 (-42.1 to 21.0) | 0.51 |  | -7.9 (-39.0 to 23.3) | 0.62 |  |
|  | Severe | -39.8 (-75.63 to -3.9) | 0.03 |  | -40.0 (-75.7 to -4.3) | 0.03 |  | -40.5 (-77.3 to -3.7) | 0.03 |  | -41.2 (-77.3 to -5.1) | 0.03 |  |
|  |  |  |  |  |  |  |  |  |  |  |  |  |  |
| **Imputed data (n=100)** |  |  |  |  |  |  |  |  |  |  |  |  |  |
| Δ Knee quadriceps strength (Nm/kg) |  |  |  | 0.23 |  |  | 0.23 |  |  | 0.22 |  |  | 0.30 |
|  | Mild | -1.6 (-25.7 to 22.6) | 0.90 |  | -0.3 (-25.3 to 24.7) | 0.98 |  | -0.8 (-26.2 to 24.6) | 0.95 |  | 7.04 (-18.8 to 31.9) | 0.59 |  |
|  | Moderate | -14.0 (-37.0 to 9.0) | 0.23 |  | -14.9 (-37.7 to 8.0) | 0.20 |  | -14.9 (-38.2 to 8.5) | 0.21 |  | -16.46 (-38.6 to 5.6) | 0.14 |  |
|  | Severe | -21.4 (-37.6 to -5.2) | 0.01 |  | -21.4 (-37.4 to -5.5) | 0.10 |  | -21.4 (-37.5 to -5.4) | <0.01 |  | -22.10 (-37.4 to -6.8) | 0.01 |  |
| Δ Knee hamstring strength (Nm/kg) |  |  |  | 0.18 |  |  | 0.18 |  |  | 0.16 |  |  | 0.24 |
|  | Mild | -1.2 (-38.4 to 36.1) | 0.95 |  | 2.5 (-35.7 to 40.7) | 0.90 |  | 1.8 (-37.0 to 40.7) | 0.93 |  | -8.47 (-47.6 to 30.6) | 0.67 |  |
|  | Moderate | -8.2 (-44.2 to 27.7) | 0.65 |  | -8.0 (-43.6 to 27.7) | 0.66 |  | -9.0 (-45.7 to 27.8) | 0.63 |  | -5.10 (-40.4 to 30.2) | 0.77 |  |
|  | Severe | -34.1 (-76.0 to 7.9) | 0.11 |  | -33.2 (-75.2 to 8.8) | 0.12 |  | -32.2 (-75.2 to 10.7) | 0.14 |  | -33.81 (-73.5 to 5.9) | 0.09 |  |
| ^1^ adjusted for gender, age  ^2^ adjusted for gender, age, exercise group, baseline strength  ^3^ adjusted for gender, age, exercise group, baseline strength, change in pain (VAS) | | | | | | | | | | | | | |
